# Mapping the Human Brain in Frequency Band Analysis of Brain Cortex Electroencephalographic Activity for Selected Psychiatric Disorders

**DOI:** 10.3389/fninf.2018.00073

**Published:** 2018-10-24

**Authors:** Grzegorz M. Wojcik, Jolanta Masiak, Andrzej Kawiak, Lukasz Kwasniewicz, Piotr Schneider, Nikodem Polak, Anna Gajos-Balinska

**Affiliations:** ^1^Department of Neuroinformatics, Faculty of Mathematics, Physics and Computer Science, Institute of Computer Science, Maria Curie-Sklodowska University in Lublin, Lublin, Poland; ^2^Neurophysiological Independent Unit of the Department of Psychiatry, Medical University of Lublin, Lublin, Poland

**Keywords:** electroencephalography, sLORETA, psychiatric disorders, frequency band analysis, biomarkers, working memory, DIGITS

## Abstract

There are still no good quantitative methods to be applied in psychiatric diagnosis. The interview is still the main and most important tool in the psychiatrist work. This paper presents the results of electroencephalographic research with the subjects of a group of 30 patients with psychiatric disorders compared to the control group of healthy volunteers. All subjects were solving working memory task. The digit-span working memory task test was chosen as one of the most popular tasks given to subjects with cognitive dysfunctions, especially for the patients with panic disorders, depression (including the depressive phase of bipolar disorder), phobias, and schizophrenia. Having such cohort of patients some results for the subjects with insomnia and Asperger syndrome are also presented. The cortical activity of their brains was registered by the dense array EEG amplifier. Source localization using the photogrammetry station and the sLORETA algorithm was then performed in five EEG frequency bands. The most active Brodmann Areas are indicated. Methodology for mapping the brain and research protocol are presented. The first results indicate that the presented technique can be useful in finding psychiatric disorder neurophysiological biomarkers. The first attempts were made to associate hyperactivity of selected Brodmann Areas with particular disorders.

## Introduction

Dense array electroencephalographic amplifiers can be considered as a reasonable alternative for magnetic resonance imaging (MRI) thanks to their better temporal resolution (Tohka and Ruotsalainen, 2012) and application of algorithms like standardized low-resolution brain electromagnetic tomography (sLORETA) (Pascual-Marqui et al., 1994; Pascual-Marqui, 2002) that allow to compute and then visualize brain cortex activity in resolution similar to that obtained from computer tomography with temporal precision enabling observation of cortical responses appearing right after given stimuli. Such techniques of imaging are widely used in laboratories of experimental psychology and more and more often for research in neuroscience. What is more—the electroencephalography (EEG) is much cheaper than other methods, practically non-invasive and the subject or patient can participate in the experiment without any special requirements (Tohka and Ruotsalainen, 2012). Electroencephalographic techniques find applications in adult psychiatry (Sand et al., 2013). Electrophysiological methods have developed in recent decades (Kamarajan and Porjesz, 2015; Martínez-Rodrigo et al., 2017). Recently there has been a rapid advance in therapeutic use of Brain-Computer Interfaces (BCI) in which the acquisition of electrical activity of selected areas of brain cortex plays the main role (Mikołajewska and Mikołajewski, 2012, 2013, 2014; Teruel et al., 2017) and Event-Related Potentials (ERP) and other evoked potentials can lead not only to explanation of psychological behaviors in particular situations (Kotyra and Wojcik, 2017a,b) but also to finding some biomarkers characteristic of psychiatric disorders (Chapman and Bragdon, 1964; Sutton et al., 1965; Campanella, 2013; Golonka et al., 2017). Together with the development of neurocomputing, neuroinformatics and artificial intelligence a lot of new tools and possibilities appeared and made their use possible for a wide range of classification tasks in biomedical engineering (Ogiela et al., 2008; Szaleniec et al., 2008, 2013) or brain functions simulations which are also a subject of our investigations (Ważny and Wojcik, 2014; Wojcik and Ważny, 2015). The computational approach can explain some behavior characteristic of complex systems (Wojcik et al., 2007; Wojcik and Kaminski, 2008; Wojcik and Garcia-Lazaro, 2010) or even investigate the influence of electrophysiological parameters of single cells on the dynamics of the whole simulated system (Wojcik and Kaminski, 2007; Wojcik, 2012) but it still cannot explain such complicated phenomena like psychiatric disorders or mechanisms responsible for variety of syndromes (e.g., burn-out; Chow et al., 2018).

In current psychiatry, the interview is still a main tool for diagnosis. This is the clinical interview that in most cases determines the psychiatrist to choose the optimal method of treatment. So it is easy to imagine that sometimes the treatment is not as optimal as it should be. As far as the EEG-based diagnosis support for psychiatry is concerned some works were presented by John in the late eighties (John et al., 1988) using spectral analysis, however, the source localization algorithms technique did not developed yet at that time.

There are different types of representation for EEG activity but one of the oldest and most popular is its characteristic in frequency bands that describe rhythmical nature of its waves. Thus there are a few bands that in the literature (Niedermeyer and da Silva, 2005) are described as follows: δ—delta band (less than 4 Hz), θ—theta (4–7 Hz), α—alpha (8–15 Hz), β—beta (16–31 Hz), γ—gamma (more than 31 Hz) and sometimes μ—mu (8–12 Hz).

The aim of the research presented in this paper was to prepare the protocol and methodology for mapping the brain in five bands of EEG spectrum using the sLORETA algorithms. Source localization, among other algorithms, seems to be one of the most appropriate approaches for finding biomarkers in EEG signals. The method used in a wide range of research—from neurodegenerativie diseases (Wu et al., 2014) to attention-deficit-hyperactivity disorder (ADHD) (Mann et al., 1992) proves its effectiveness also in frequency band analysis (Moretti et al., 2004; Saletu et al., 2010) and on the electrophysiological landscape it was applied in psychiatry even by one of its pioneers in Pascual-Marqui et al. (1999). This is the initial stage of the research and this technique is believed to be crucial for finding psychiatric disorder neurophysiological biomarkers.

For this contribution the brain activity of a group of 30 patients with selected psychiatric disorders was measured using 256-channel dense array EEG. The sLORETA algorithm was applied in alpha, beta, gamma, delta and theta EEG frequency bands. These results were compared with those obtained for the participants of a control group both doing working memory span task.

## Materials and methods

**Figure 1:**
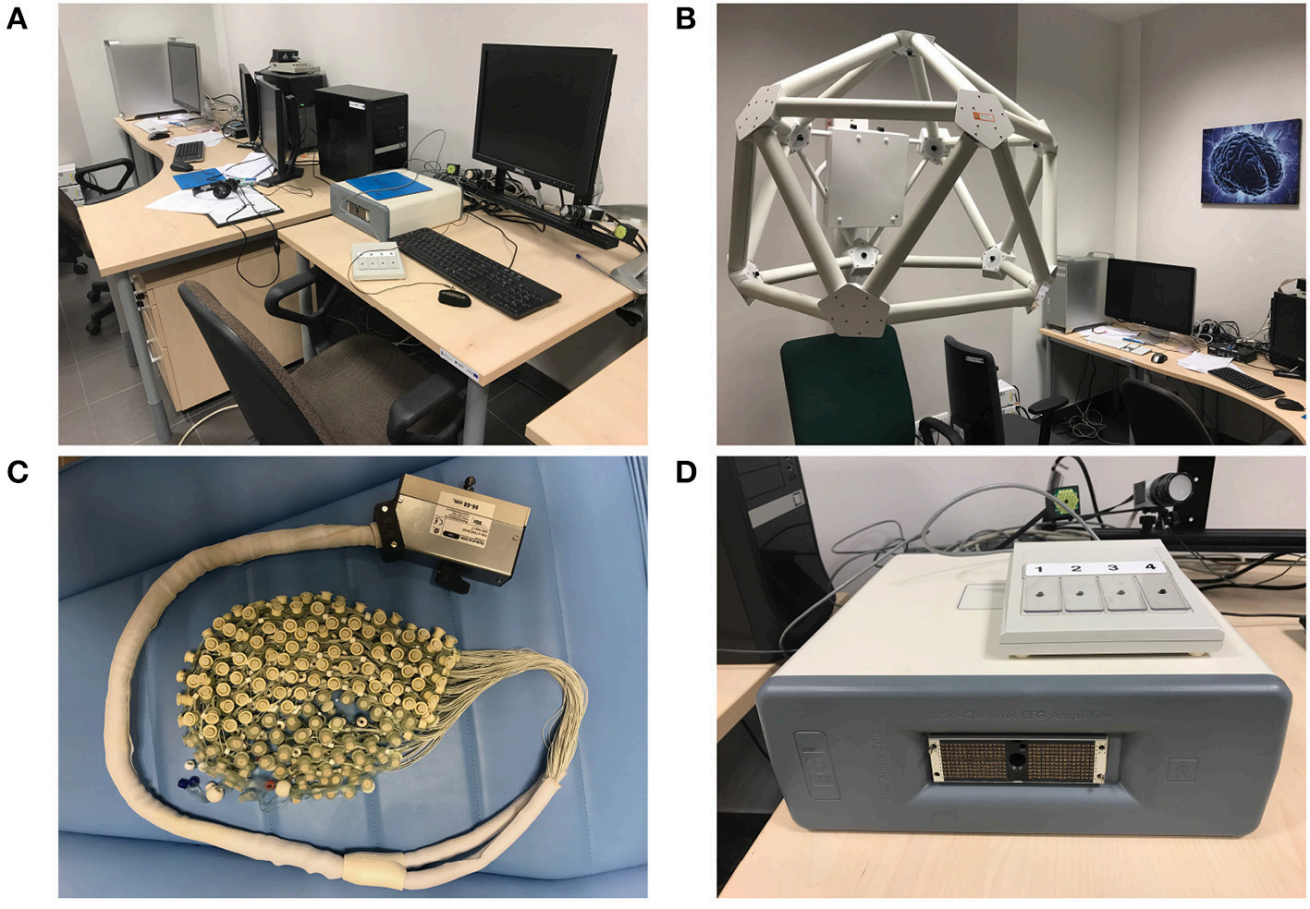
EEG Laboratory in the Department of Neuroinfomatics. From the top-left corner clockwise: **(A)** general lab view, **(B)** GPS photogrammetry station, **(D)** 256-channel dense array amplifier with response pad, **(C)** Geodesic Sensor Net with 256 electrodes.

The EEG Laboratory (see Figure 1) in the Department of Neuroinformatics is equipped with the dense array amplifier able to record the brain electrical activity with 500 Hz frequency through 256 channels HydroCel GSN 130 Geodesic Sensor Nets provided by EGI^1^. In addition, there was used the Geodesic Photogrammetry System (GPS) which owing to 11 cameras put in its corners makes a model of subject brain based on its calculated size, proportion and shape and then puts all computed activity results on this model with very good accuracy. The amplifier works with the Net Station 4.5.4 software, GPS under control of the Net Local 1.00.00 and GeoSource 2.0. The gaze calibration, eye blinks and saccades elimination are obtained owing to the application of eye-tracking system operated by SmartEye 5.9.7. The ERP experiments are designed in the PST e-Prime 2.0.8.90 environment ^2^.

The cohort of 30 patients, 21 males and 9 females (avg. age 28.1, s.d. 12.4) diagnosed a wide range of psychiatric disorders classified in ICD-10 as: 1 × F20 (Schizophrenia), 2 × F31 (Bipolar affective disorder), 5 × F32.1 (Moderate depressive episode), 3 × F40 (Phobic anxiety disorders), 12 × F41 (Other anxiety disorders, Panic disorder), 2 × F42 (Obsessive-compulsive disorder – among patients with F84.5), 2 × F51.1 (Non-organic hypersomnia), 5 × F84.5 (Asperger syndrome). They were not treated earlier and participated in the experiment before taking the first dose of suggested medications. The results were compared with those of the participants from the control group of 30 healthy volunteers, males (avg. age 22.4, s.d. 1.7). In fact, about 30% more subjects both from patients and control groups have been investigated, because all those for whom the signal was too noisy or incomplete had to be eliminated.

One of the tests that are quite often used in experimental psychology is the digit-span task (Jones and Macken, 2015). There is a handful of literature reviews and our own studies which show that different cognitive functions in patients with psychiatric disorders are not as effective as among healthy representatives of populations (Trivedi, 2006). People with mental disorders often suffer from working memory dysfunctions and the digit-span task is used then to measure their level. The digit-span task is very popular in the investigations of subjects with phobias, panic disorders, depression (including the depressive phase of bipolar disorder) and schizophrenia (Alves et al., 2013; Zhou and Ni, 2017) and in our cohort of patients including 23 with mentioned above diagnoses. It was natural that to examine the influence of Asperger syndrome and insomnia on working memory parameters – as the rest of t patients from our cohort suffered from these disorders.

Thus the digit-span task (DIGITS) was used in order to determine subject's working memory capacity^3^. The experiment gives sets of 6 trials on a set of digits that starts with a length of 3 and goes up or down depending on subject's performance (more than 3 correct makes the number of digits increase, less than 2 makes it decrease). The longest sequence of digits is 8. There are 5 sequences of digits in each trial.

**Figure 2:**
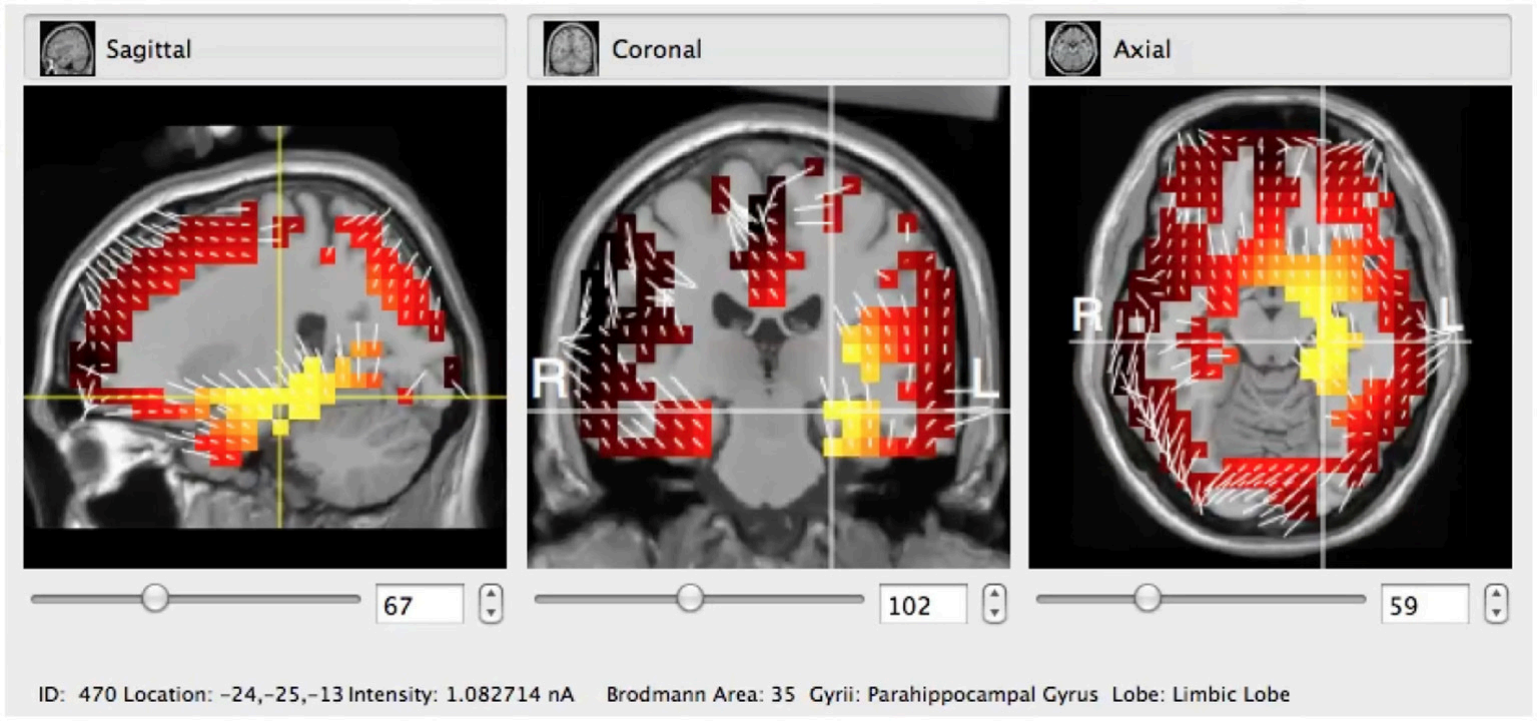
Typical visualization of sLORETA algorithm applied to the GeoSource pre-processed raw EEG signal in coronal, sagital, and axial cross-sections. Here the BA35 (Parahippocampal Gyrus, Limbic Lobe) is indicated.

**Figure 3:**
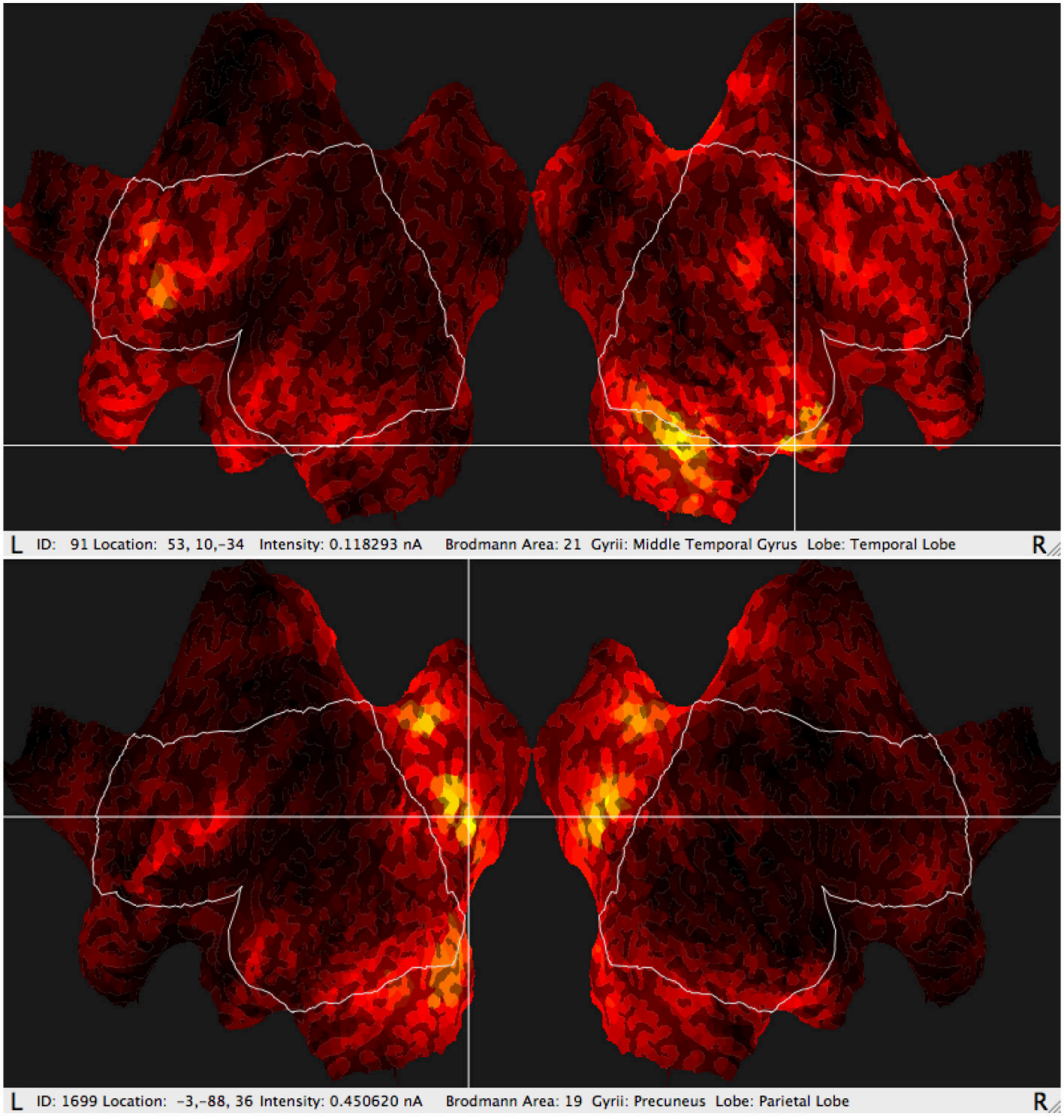
Typical results of GeoSource BA activity visualization on the brain cortex so-called Flat Map. Increase of activity in BA21 (Middle Temporal Gyrus, Temporal Lobe) and BA19 (Precuneus Lobe, Parietal Lobe) is indicated.

Then using an appropriate band filtering tool provided by the Net Station software, the signal to analysis in the GeoSource (see Figures 2, 3) was prepared. After applying the sLORETA algorithm to the signal preprocessed in the above mentioned it was possible to indicate precisely the Brodmann Area (BA) of brain cortex that was most active during the experiment in particular subjects.

The sLORETA implemented in our Laboratory was the most standard version of the algorithm broadly described in the Brain Source Localization Using EEG Signals chapter of Nidal and Malik (2014). The sLORETA method is based on the assumption of the standardization of the current density. Its implication is that not only the variance of the noise in the EEG measured signal is taken into account but also that the biological variance in the actual signal is considered (Goldenholz et al., 2009; Nidal and Malik, 2014). This biological variance is taken as independently and uniformly distributed across the brain which results in a linear imaging localization technique having exact, zero-localization error (Goldenholz et al., 2009; Nidal and Malik, 2014). Perfect and detailed comparison of different variations of LORETA is presented in Nidal and Malik (2014).

The procedure of estimating the most active BAs was as follows: after the signal acquisition, the subject was photographed using 11 cameras in the GPS. Then the GeoSource software calculated the activity of particular BAs (in nanoamperes) varying in time and this varying activity together with its corresponding BAa were saved in the appropriate list. Then our scripts chose the activity that was the highest in a given short interval of time. There was considered not only the maximum value of the electrical current of a given BA in a given interval but also the time in the range of the interval in which this activity was maintained. Thus, in other words, the maximum activity was equivalent to the electric charge that flew through the given area.

Measuring the electric charge flowing thorough the selected BA can shed some light on dynamic activity analysis and seems to be better than typical amperage-based interpretations. Together with the frequency band analysis it creates a kind of quantitativeness in the quantitative analysis of biomedical signal source localization based analysis.

The time interval in which the BA activity was calculated was set to 5 ms and there was chosen 800 ms segmentation (each segment starting with the stimuli) for signal averaging.

BA1, BA2, and BA3 were eliminated from our considerations as they are part of Primary Somatosensory Cortex (S1) which must have been highly active (and in fact reported by our algorithms) because of the subject's fingertips contact with the keyboard during the experiment.

**Figure 4:**
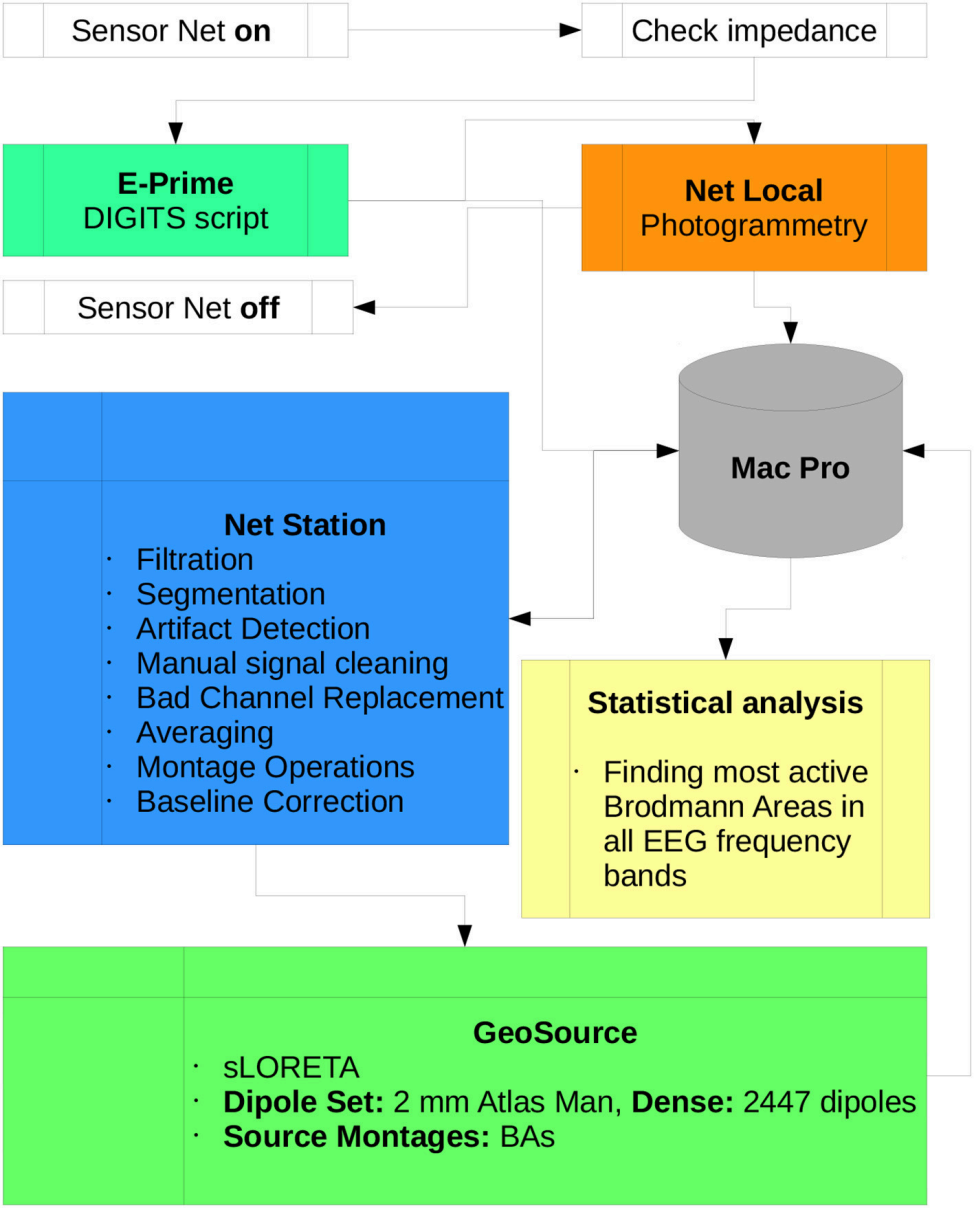
Diagram of the DIGITS research protocol proposed in this paper. All scripts used for preprocessing data in Net Station and postprocessing in GeoSource are listed. Participation of the subject in the experiment begins when the Sensor Net is put on and ends when it is taken off. All data is collected by the Mac Pro workstation which is the central part of the lab. Statistical analysis, finding the most active BAs in each of α, β, γ, δ, and θ frequency bands can be conducted on other machines.

The scheme of the methodology and research protocol are presented in Figure 4.

The software used to conduct discussed experiments was provided by EGI. In the Net Station package there are all scripts shown in Figure 4 implemented as the so-called Waveform Tools. The details of algorithms used in the preprocessing and postprocessing phases of experiment are described in detail in Electrical Geodesics (2003). Source Localization and algorithms used in photogrammetry Net Local are also described in the EGI Lab documentation (Electrical Geodesics, 2009, 2011), respectively.

## Results

Some EEG biomarkers are assumed to appear and leave a kind of particular disorder fingerprint in the selected EEG band. In addition alpha, beta, and theta bands are important for as above computing the engagement index (Lubar et al., 1995; Pope et al., 1995; Chaouachi et al., 2010) and can play a significant role in the different manifestations of psychiatric disorders as the activity observed in those bands comes from different regions of the brain representing, in fact, different cognitive processing abilities.

The results for the patients are presented in Table 1 and for the control group in Table 2.

**Table 1:** Most active BA in particular subjects of patients group during the digit-span task experiment in the alpha, beta, gamma, delta, and theta EEG bands.

**No**.	**Diag**.	**α**	**β**	**γ**	**δ**	**θ**
1	F20	R32	R32	LA	R32, L28	R9
2	F31	R9	LA	R9	R33	R9
3	F31	R9	R33, R9	S1	R33	R9
4	F32.1	LA	L45	R9	R33	R9
5	F32.1	L27, R9	L27	R9	L27	R9, L27
6	F32.1	R9	L27	R9	L27	R9
7	F32.1	R33	R33, R9	L27, R9	R33, L27	R9, L27
8	F32.1	R9	R9, L45	S1	R33	R9
9	F40	LA	R4	S1	R7	LA
10	F40	R9	R9, L45	R9	LA, R9	R9
11	F40	R9	R7	R23	R7	R9
12	F41	R9	L27	S1	L27	R9
13	F41	R9	R9, L45	R9	R34	LA
14	F41	R9, L45	L45	LA	L45	R9
15	F41	L27, R41	L27, R41	LA	L27, R33	L27, R41
16	F41	R27	R27	R9	L24, R4	R9
17	F41	R7	R7	R9, LH	R4	R7
18	F41	R9	R33, R44	L36	R33	LH
19	F41	L27, R41	L27	R41	L27	L27
20	F41	R9	LA, R8	R9	R7	R9
21	F41	R13, R27, R34	R33, R34	R13, R34	R33, R34	R34
22	F41	L45	L45	L45, LA	L45	L45
23	F41	R9	R7, R9	L29	S1	L27
24	F51.1	R9	L27	R41	L27	R9, LH
25	F51.1	L45	R41	LA	L27	R9
26	F84.5	L9	R13, R33	S1	LA, L27	L7
27	F84.5	R9	R36, L24	R9	R36	R9
28	F84.5, F42	R24	R24	L45, LA	R24	LH
29	F84.5, F42	LH, LA	LA, R27	R9	L45	R9
30	F84.5	L45	L45, R44	L27, L37, L43	L27	L45, L37

*“A” indicates Amygdala, “H” for Hippocampus areas. S1 the areas of Primary Somatosensory Cortex. L and R the left and right hemispheres, respectively. Example: L45 is the left hemisphere BA45 and R41 is the right hemisphere BA41, LA—left hemisphere of the Amygdala. The most active BA were manually counted for particular disorders. For detail see Discussion section in text*.

**Table 2:** The most active BA in particular subjects of control group during the digit-span task experiment in the alpha, beta, gamma, delta and theta EEG bands.

**No**.	**α**	**β**	**γ**	**δ**	**θ**
1	R9	R7, R33	R46	"R33, R34	R9, R33
2	R9	R9, LA	S1	R33	R9
3	R9	R9	R9	L46	R9
4	L23, R9	L27, R41, R33	L33, R33	L18, L24	R44, L33, L45
5	R9	L27, R36, L24	L27, R8	L36, L27	R9
6	R9	L45, R9	LA, L24	L45, L46	R9
7	R41	R41	R27	L27	R9
8	R9	R33, R33	S1	L27	R9
9	R9	L27	R4	L27	R9
10	R32	R32	R9	L27	R9
11	R9	L27, R28	R9	L27	R9
12	R33, L27	R33, R41, L27	R36	LH	L24
13	R9, R24	R41, LA, R11	R9	R11, R7, R24	R9
14	R9	R9	R9, LA	R9	R9
15	R9	R7, LA	LA, R4	R7	R9
16	R9	R9, L18	S1	L27, R7	S1
17	R7	R7	LA	R7	LA
18	R9	LA	R9	LA	R9
19	R9	R33, R7	R9	R7	R9
20	L13	L13, L27	R9	L27	R9
21	R9,	R33	R9	R33	R9
22	L27, R9	L27	L27	L27	L27
23	LA	R44	R44	R44	LA
24	R9, L27, R7	L27	R27	L27	R9
25	LA, R27	LA	S1	LA	LA
26	S1	S1	S1	S1	S1
27	R9	R36, R9	R9	L24, L36	R9
28	R9	L45	S1	L27	R9
29	R9	LA, R9	R9	LA	R9
30	R9	LA	L44	S1	R9

*“A” indicates Amygdala, “H” the Hippocampus areas. S1 the areas of Primary Somatosensory Cortex. L and R the left and right hemispheres, respectively. Example: L27 is the left hemisphere BA27 and R33 is the right hemisphere BA33, RH—right hemisphere of the Hippocampus*.

Indeed, as one can see in Tables 1, 2 it was possible to indicate the Brodmann Areas that were most active in each of five bands during the working memory task completed by all subjects of the experiment. In the subjects where the eliminated S1 was the only high active part of the cortex, S1 was put in both tables. In Table 3, the names of the anatomical brain structures of the most active BA mentioned in text are presented.

**Table 3:** The names of the anatomical brain structures of the most active BA mentioned in text.

**No**.	**BA**	**Anatomical brain structure**
1	BA9	Dorsolateral prefrontal cortex
2	BA27	Piriform cortex
3	BA33	Anterior cingulate cortex
4	BA34	A part of the entorhinal area and the superior temporal gyrus
5	BA41	Anterior transverse temporal area
6	BA45	Pars triangularis of the inferior frontal gyrus

In Table 1 one can see that among the largest subset of patients group, 12 suffered from F41—Panic disorders and 5 from F32.1— Depression, 5 had the Asperger syndrome. At present it is, of course, impossible to talk about the psychiatric atlas of the human brain having such a small trial and so large group of disorders defined in ICD-10. However, one can read from Table 1 that when compared to the control group in Table 3:

- Among patients with F41 the increased activity in right BA33, BA34, BA41 in the auditory cortex and left Amygdala can be observed.
- Among patients with F32.1 the increased activity of right BA33, especially in δ band ought to be noted. Please note that BA33 is also very active in the bipolar affective patients F31.
- Among patients with F84.5 hyperactive are the left BA45 and left Amygdala.

As above that BA33 is responsible for the modulation of emotional responses (Posner and DiGirolamo, 1998; Bush et al., 2000; Nieuwenhuis et al., 2001).

Similarly, BA45 is associated with semantic tasks and working memory (Buckner, 1996; Gabrieli et al., 1998).

Overactive BA9 in both patients and control groups is engaged in management of cognitive processes (Elliott, 2003), including working memory (Barbey et al., 2013), cognitive flexibility (Monsell, 2003), and planning (Chan et al., 2008). This supports the evidence for our properly working experimental set-up.

It is interesting that also BA27 associated with the sense of smell (Howard et al., 2009) is active in most of the subjects as well.

It may be also interesting to specify the role of auditory cortex hyperactivity among some patients and interesting EEG experiments were presented (e.g., in Martínez-Rodrigo et al., 2017).

## Discussion

We have proposed the research protocol and methodology for investigation of working memory in patients with selected psychiatric disorders. The sLORETA algorithms and source localization were chosen to find highly active areas of brain cortex during the experimental task. Profound analysis of cortical activity in five EEG frequency bands allows to us look into the brain dynamics in different spectral ways just as we are used to looking at the Universe and its stars.

Having so many mental disorders defined in ICD-10 one can imagine the size and complexity of the job that must be done to build a good atlas for psychiatrists. Designing new experiments the attempt will me made to choose the most appropriate tests for particular disorders and apply other variations of sLORETA described in Nidal and Malik (2014). Building appropriate statistical groups of patients with a given disorder, untreated, in similar age ranges and distinguished for genders seems to be a task for many years of research.

However, these first results make us hope that it is really possible to find association of selected Brodmann Areas activity with psychiatric disorders. As it was mentioned above—we will need a huge number of untreated patients suffering from each of disorders that we want to map and if we are right—finally it will lead to the creation of Atlas which can throw lights on modern psychiatry. Collecting the above mentioned data is also a great challenge for current neuroinformatics (Bigdely-Shamlo et al., 2016; Cavanagh et al., 2017).

Finding biomarkers for a wide range of psychiatric patients with completely different symptoms and clinical characteristics is a challenging task. The aim of this paper was not, however, to hypothesize dysfunctions of some parts of the brain in particular disorders but to show a new way in which this can be accomplished. In the group of 30 there were representatives of 8 different diagnoses. Under ideal conditions it would be proper to have c.a. 30 patients of each gender and handedness as well as in three ranges of age. That would make us record systematically the electrical activity of 1,440 patients only for these 8 disorders.

Under the above mentioned conditions it would be possible to quantify results statistically. Without an appropriate number of patients we can only show directions toward which the future research ought to be oriented.This preliminary, exploratory analysis could be a starting point for a classification or prediction strategy using large databases and data science tools, to map the brain regions involved in different psychiatric disorders and find neurophysiological biomarkers for them. Such classification and prediction study of the areas involved in different psychiatric disorders would be a much stronger and useful objective, using the presented methodology, but larger databases properly balanced and stratified are needed for that purpose. They would help answer the following questions: What are the most consistent areas within groups or type of disorder? What are the the differences between activation maps and amplitudes between groups? Is the variability in active areas different between groups? Would the results be the same if using other inverse methods?.

And above all results and implications coming from such works can increase the comfort of life of many people notably.

## Ethics statement

This study was carried out in accordance with the recommendations of Guidelines for Good Clinical Practice (GCP). The protocol was approved by the Medical University of Lublin Bioethical Commission. All subjects gave written informed consent in accordance with the GCP. Permission No. KE-0254/138/2015 given by Medical University of Lublin Bioethical Commission on May 28th, 2015.

## Author contributions

GW: project idea and coordination, experiment design, subjects' recruitment, and results' interpretation. JM: project idea, experiment design, subjects' recruitment, psychiatric diagnosis, and results' interpretation. AK: work in laboratory, cleaning signal, computations, and statistical analysis. PS and LK: statistical analysis, writing scripts, work in laboratory, cleaning signal. NP and AG-B: work in laboratory.

### Conflict of interest statement

The authors declare that the research was conducted in the absence of any commercial or financial relationships that could be construed as a potential conflict of interest.

## Footnotes

^1^Electrical Geodesic Systems, Inc., 500 East 4th Ave. Suite 200, Eugene, OR 97401, USA.

^2^Psychology Software Tools, Inc.PST, Sharpsburg Business Park, 311 23rd Street Ext., Suite 200, Sharpsburg, PA 15215-2821 USA.

^3^The e-Prime scripts for this test can be found at https://step.talkbank.org.
